# Effects of Sethoxydim on Root Morphology and Rhizosphere Microecology of Foxtail Millet Seedlings

**DOI:** 10.3390/plants15182788

**Published:** 2026-09-11

**Authors:** Qi An, Manqing Luo, Shuo Li, Suqi Shang, Jianhong Ren, Xiaorui Li, Xi’e Song, Huiling Du, Xiangyang Yuan, Chunyan Hu, Shuqi Dong

**Affiliations:** 1 College of Agriculture, Shanxi Agricultural University, Jinzhong 030800, China; a7n7q7i@163.com (Q.A.); leesure2025@163.com (S.L.); s1883406@163.com (S.S.); lixiaorui@sxau.edu.cn (X.L.); sxndsxe@163.com (X.S.); 2 College of Plant Protection, Shanxi Agricultural University, Jinzhong 030800, China; luomanqing728@163.com; 3 College of Life Sciences, Shanxi Agricultural University, Jinzhong 030800, China; renjh@sxau.edu.cn; 4 Special Orphan Crops Research Center of the Loess Plateau, MARA, Shanxi Agricultural University, Jinzhong 030800, China; duhuiling66@163.com; 5 Shanxi Institute of Functional Agriculture, Shanxi Agricultural University, Jinzhong 030800, China

**Keywords:** foxtail millet, sethoxydim, root morphology, rhizosphere microorganism, soil enzyme activity

## Abstract

Sethoxydim, a cyclohexanedione herbicide inhibiting acetyl-CoA carboxylase (ACCase), is widely used for post-emergence control of graminaceous weeds in foxtail millet (*Setaria italica* (L.) Beauv.) fields. This study investigated under field conditions the effects of sethoxydim on root morphology and rhizosphere microecology using two cultivars with contrasting tolerance: the relatively tolerant hybrid Zhangzagu 10 and the relatively sensitive conventional Zhonggu 19. Previous studies on sethoxydim in foxtail millet have focused primarily on shoot physiology, whereas integrated assessments of root morphology, soil enzyme activity, and rhizosphere microbiome remain scarce. Sethoxydim was applied at 112.5, 225.0, or 337.5 g a.i./hm^2^ (0.5×, 1×, and 1.5× the recommended dose), with water as control. Root morphology and rhizosphere enzyme activities were measured at 0, 7, 14, 21, and 28 days after application, and bacterial and fungal communities were analyzed by high-throughput sequencing. Sethoxydim inhibited root growth in both cultivars, but Zhonggu 19 was consistently more sensitive. At 337.5 g a.i./hm^2^, root length, surface area, diameter, and volume were markedly reduced in Zhonggu 19, whereas Zhangzagu 10 showed milder inhibition and even slight stimulation at lower doses. Rhizosphere urease, sucrase, and catalase activities initially decreased then recovered. Sethoxydim altered α-diversity and community structure of rhizosphere bacteria and fungi. These preliminary findings suggest that sethoxydim may be tentatively applied at or below the recommended dose (≤225.0 g a.i./hm^2^) under similar field conditions, particularly for tolerant cultivars such as Zhangzagu 10, though confirmation through multi-year trials is required.

## 1. Introduction

Foxtail millet, an important minor cereal crop in China, exhibits exceptional drought tolerance, adaptability to poor soils, and strong environmental resilience, making it indispensable in dryland agriculture [1,2,3]. Foxtail millet grows slowly during the seedling stage, exhibiting weak competitive ability and high susceptibility to weed competition [4]. Untimely weed control severely restricts crop establishment and ultimately leads to significant yield losses [5]. Therefore, effective weed management is essential for ensuring stable and high yields in foxtail millet production [6].

Weeds in foxtail millet fields include both graminaceous species, such as green foxtail (*Setaria viridis* (L.) P. Beauv.), barnyard grass (*Echinochloa crus-galli* (L.) P. Beauv.), and wild panicum (*Panicum miliaceum* L. var. *ruderale* Kitag.), and broadleaf weeds, such as *Chenopodium* spp., *Polygonum* spp., and *Amaranthus* spp. [7]. Current herbicide options for broadleaf weed control include atrazine, 2,4-D butyl ester, sulfometuron-methyl, and bromoxynil [8]. For graminaceous weeds, farmers commonly use sethoxydim, prometryn, S-metolachlor, and acetochlor [9]. However, herbicide phytotoxicity remains a significant concern in practical applications. Some herbicides show poor selectivity. For example, improper application or excessive rates of atrazine during the seedling stage can cause severe phytotoxicity [10]. This includes growth inhibition, oxidative damage, and disrupted photosynthesis. Despite these risks, chemical weed control remains indispensable due to its high efficiency and operational convenience [11].

Sethoxydim belongs to the cyclohexanedione class of herbicides. It is a selective systemic post-emergence herbicide [12]. Its mode of action involves inhibition of acetyl-CoA carboxylase (ACCase) activity in plants. At recommended doses (225 g a.i./hm^2^), sethoxydim causes transient damage to foxtail millet [13]. Plants can recover through self-regulation of physiological functions [14]. However, improper application or excessive rates during the seedling stage cause severe phytotoxicity. Tolerance varies significantly among foxtail millet cultivars [15].

Herbicide stress disrupts reactive oxygen species (ROS) homeostasis, inhibiting root growth and altering rhizosphere exudate composition to recruit beneficial microorganisms [16,17,18,19]. Rhizosphere enzyme activity and microbial communities, which are critical for nutrient cycling and plant stress adaptation [20,21,22], are significantly affected by herbicides [23,24]. Integrating root morphology, enzyme activity, and microbiome analysis is therefore essential for assessing herbicide stress.

Previous studies on sethoxydim in foxtail millet have focused primarily on shoot physiology and photosynthesis. Studies systematically analyzing stress effects and cultivar differences through root morphology, soil enzyme activity, and rhizosphere microbial communities remain scarce. In this study, Zhonggu 19 and Zhangzagu 10 were used as experimental materials, and different concentrations of sethoxydim were applied to determine changes in root morphological parameters, rhizosphere soil enzyme activities, and rhizosphere bacterial and fungal communities in millet seedlings. The purpose of this study was to explore the effects of sethoxydim on the physiological characteristics and rhizosphere ecological processes of foxtail millet seedlings, and to compare the response differences of different varieties to sethoxydim stress, so as to provide an important theoretical basis for the safe application of sethoxydim in foxtail millet fields and the screening of herbicide-resistant varieties. Based on the above background, we formulated the following hypotheses. (1) Sethoxydim exposure would inhibit root morphological development more severely in the sensitive cultivar Zhonggu 19 than in the tolerant cultivar Zhangzagu 10. (2) Soil enzyme activities would be differentially altered between the two cultivars, reflecting distinct rhizosphere metabolic responses. (3) The rhizosphere microbiome composition would shift under sethoxydim stress, with the tolerant cultivar harboring a more stable or beneficial microbial community structure.

## 2. Results

### 2.1. Effects of Sethoxydim Application on Root Morphology

**Figure 1:**
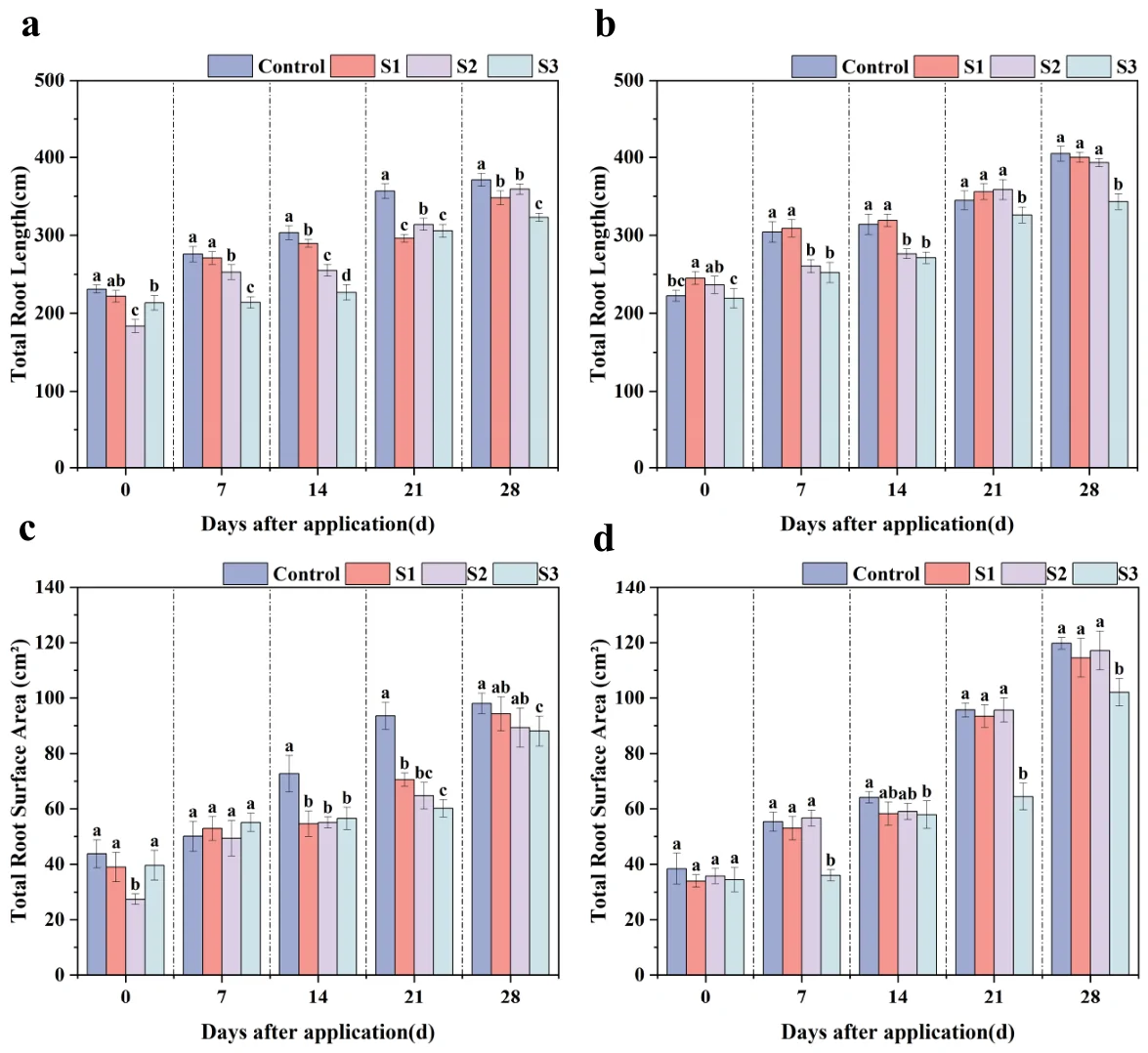

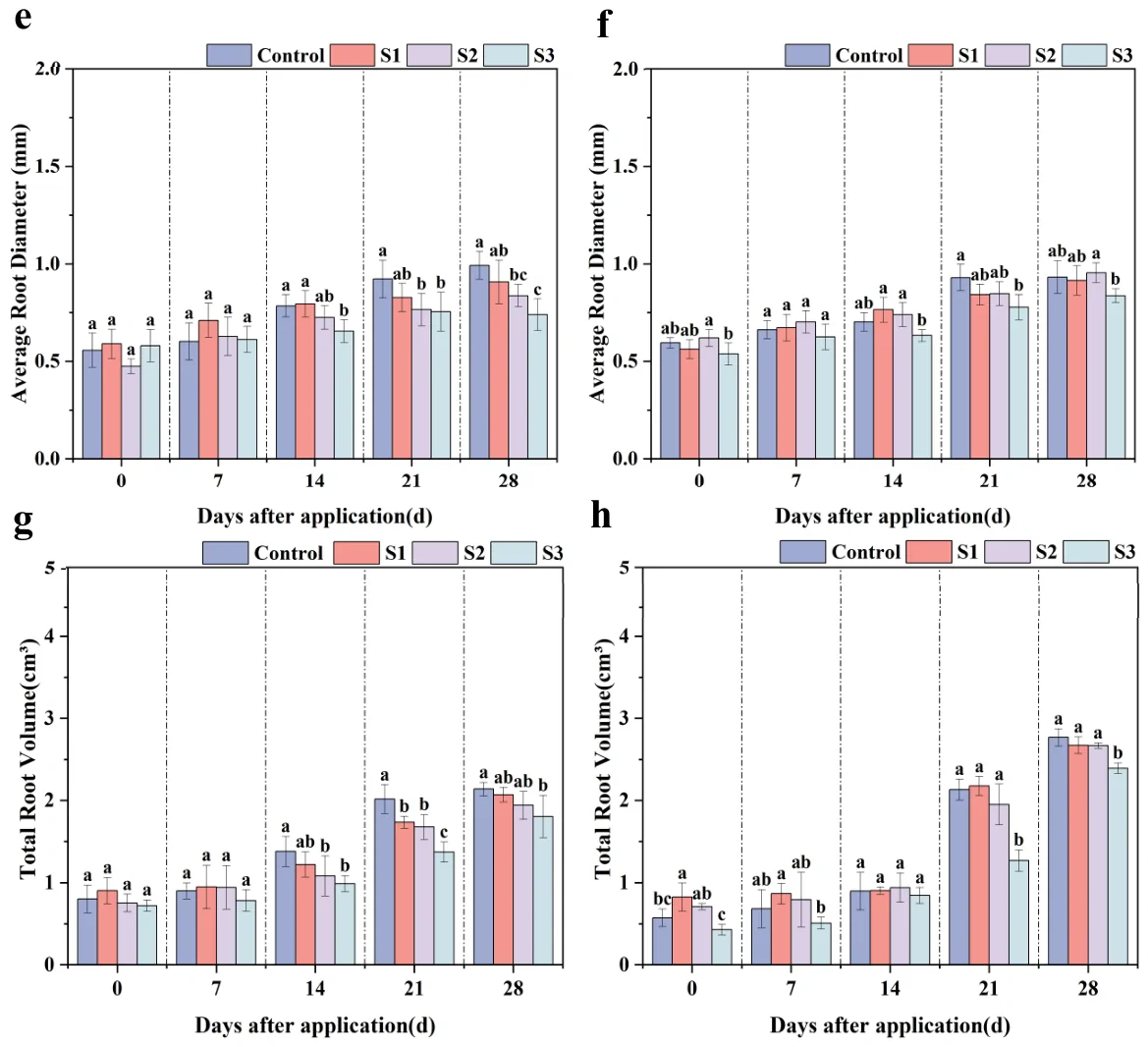
Effects of sethoxydim on root morphology in different foxtail millet cultivars. (**a**) Total root length, (**c**) total root surface area, (**e**) average root diameter, and (**g**) total root volume for Zhonggu 19; (**b**) total root length, (**d**) total root surface area, (**f**) average root diameter, and (**h**) total root volume for Zhangzagu 10. Different lowercase letters indicate significant differences among treatments at the same time point by Duncan’s multiple range test (*p* < 0.05).

One-way ANOVA indicated that herbicide dose significantly affected most root morphological parameters in both Zhonggu 19 and Zhangzagu 10 (*p* < 0.05). Cultivar-specific responses to herbicide stress were observed (Figure 1). Root parameters increased gradually across the growing period in all treatments, while the high concentration (S3) markedly inhibited root growth, with the most pronounced inhibitory effect observed under this treatment. At 28 days after application, all root parameters in Zhonggu 19 decreased relative to the control. The S3 treatment showed the greatest reduction. Total root length, surface area, average diameter, and volume decreased by 12.96% to 25.25%. In contrast, Zhangzagu 10 exhibited minimal inhibition under S1 and S2, with average root diameter even increasing by 3.13% under S2, suggesting apparently greater root plasticity under the tested conditions. This suggested a low-concentration stimulation effect. Total root length and surface area decreased by 15.22% and 14.73% under S3 relative to the control. These results suggest that Zhangzagu 10 roots may possess adaptability to sethoxydim stress. This cultivar showed relatively greater root plasticity at low and medium concentrations.

### 2.2. Effects on Rhizosphere Soil Enzyme Activity

**Figure 2:**
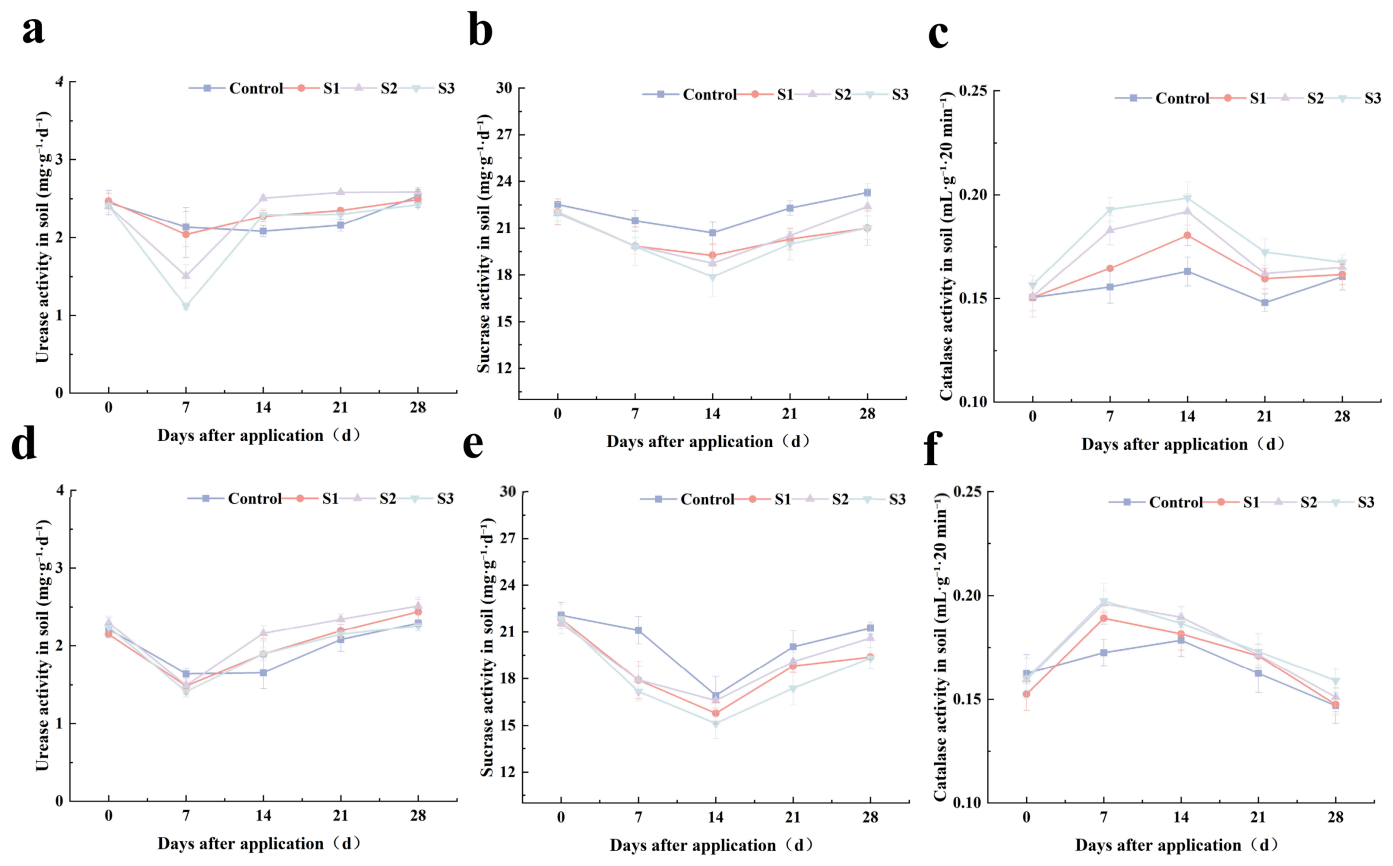
Effects of sethoxydim on rhizosphere soil enzyme activities in different foxtail millet cultivars. (**a**) Soil urease, (**b**) sucrase, and (**c**) catalase activities for Zhonggu 19, (**d**) soil urease, (**e**) sucrase, and (**f**) catalase activities for Zhangzagu 10.

One-way ANOVA revealed significant effects of herbicide dose on most rhizosphere soil enzyme activities in both cultivars (*p* < 0.05). The degree of stress varied among enzymes (Figure 2). Urease activity in both cultivars showed a decrease–increase–recovery pattern, with Zhonggu 19 exhibiting greater fluctuations and the most severe inhibition occurring at 7 days after application. Urease activity decreased by 47.42% under S3 relative to the control. At 14 days after application, all treatments exceeded the control. The S2 treatment showed the greatest increase.

Zhangzagu 10 showed significant inhibition only under S3 at 7 days after application. A transient increase occurred at 14 days after application. Activity recovered to control levels at 21 to 28 days after application. Sucrase activity was generally inhibited in both cultivars. Zhonggu 19 was inhibited primarily at 14 to 21 days after application. Activity returned to normal at 28 days after application. Zhangzagu 10 showed a significant decrease at 7 days after application. The S3 treatment remained significantly below the control at 21 to 28 days after application. This indicates a more persistent inhibitory effect of high-concentration sethoxydim on sucrase activity.

Catalase activity increased during the early stage after application. Both cultivars showed this pattern. Activity recovered during the later stage. Zhonggu 19 remained under control from 7 to 21 days after application. The S3 treatment showed the most pronounced increase. Zhangzagu 10 responded primarily at 7 days after application, with no notable differences observed thereafter. This suggests a more rapid recovery in Zhangzagu 10 under the present experimental conditions.

### 2.3. Effects on Rhizosphere Bacterial Communities

#### 2.3.1. Effects on Rhizosphere Bacterial Community Diversity

**Figure 3:**
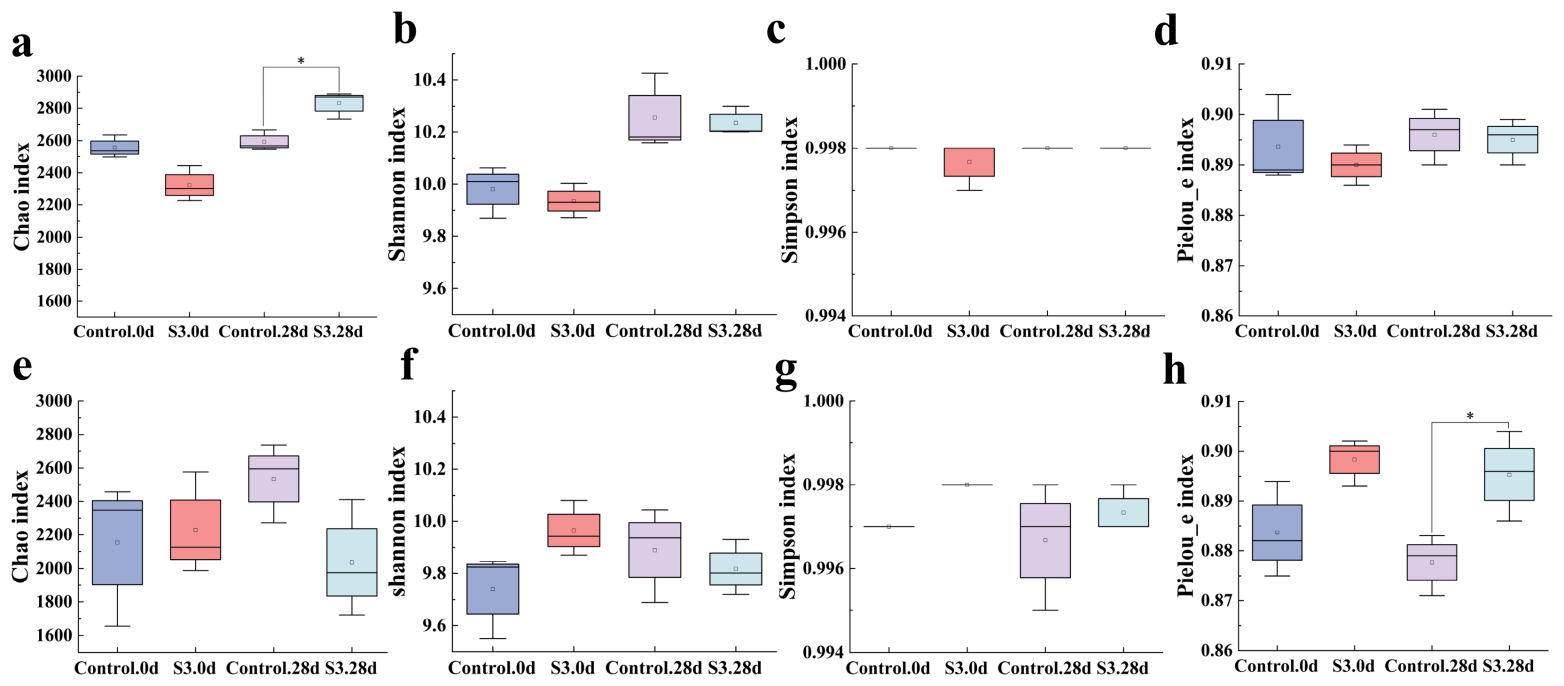
Effects of sethoxydim on rhizosphere bacterial α-diversity in different foxtail millet cultivars. (**a**–**d**) Zhonggu 19; (**e**–**h**) Zhangzagu 10. Control, water-treated plots; S3, 337.5 g a.i./hm^2^ sethoxydim treatment; 0 d, before application; 28 d, 28 days after application. Asterisks (*) indicate significant differences between treatment and control by independent-samples *t*-test (*p* < 0.05).

Independent-samples t-tests showed significant differences in bacterial α-diversity indices between the S3 treatment and the control at 28 days after application in both cultivars (*p* < 0.05). No significant differences were detected before sethoxydim application (0 d) in either cultivar (Figure 3). Significant differences in bacterial α-diversity indices were observed between cultivars at 28 days after application. Chao1 index increased significantly in Zhonggu 19 (*p* < 0.05). Shannon and Pielou’s evenness indices decreased. The Simpson index showed no significant change. Chao1, Shannon, and Simpson indices decreased in Zhangzagu 10. Pielou’s evenness index increased significantly (*p* < 0.05).

#### 2.3.2. PCoA Analysis of Rhizosphere Bacterial Communities

**Figure 4:**
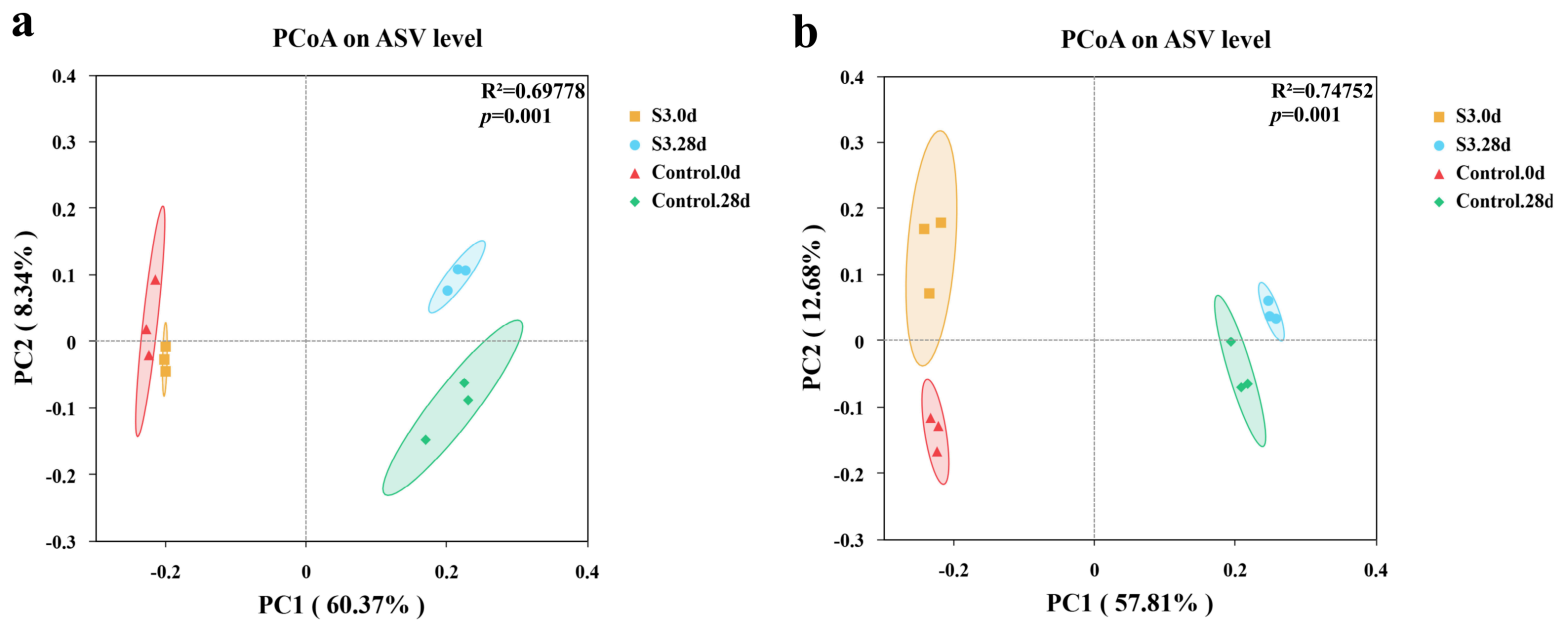
Principal coordinate analysis (PCoA) of rhizosphere bacterial communities at the ASV level based on Bray–Curtis distance. (**a**) Zhonggu 19; (**b**) Zhangzagu 10. Control, water-treated plots; S3, 337.5 g a.i./hm^2^ sethoxydim treatment; 0 d, before application; 28 d, 28 days after application. PERMANOVA (Adonis) statistics are shown in the respective panels. Pairwise comparisons between Control and S3 at 28 d were not statistically significant (*p* > 0.05).

PCoA analysis based on ASV level showed no clear separation between treatment and control groups before sethoxydim application in either cultivar (Figure 4). PERMANOVA (Adonis) revealed significant differences among all four groups in Zhonggu 19 (R^2^ = 0.698, *p* = 0.001) and Zhangzagu 10 (R^2^ = 0.748, *p* = 0.001). However, pairwise comparisons between Control and S3 at 28 d did not reach statistical significance in either cultivar (*p* > 0.05). At 28 days after application, samples from both cultivars deviated from the 0 d cluster region. PC1 and PC2 explained 60.39% and 8.33% of community variation in Zhonggu 19. The cumulative explained variation was 68.72%. PC1 and PC2 explained 57.81% and 12.68% of community variation in Zhangzagu 10. The cumulative explained variation was 70.49%. A visual tendency for separation between 28 d Control and S3 could be observed along the PC1 axis. However, this visual trend was weaker in Zhangzagu 10 than in Zhonggu 19. Although the overall rhizosphere bacterial community structure did not differ significantly between Control and S3 at 28 d, LEfSe differential-abundance analysis identified multiple microbial biomarker taxa that responded to high-dose sethoxydim stress.

### 2.4. Effects on Rhizosphere Fungal Communities

#### 2.4.1. Effects on Rhizosphere Fungal Community Diversity

**Figure 5:**
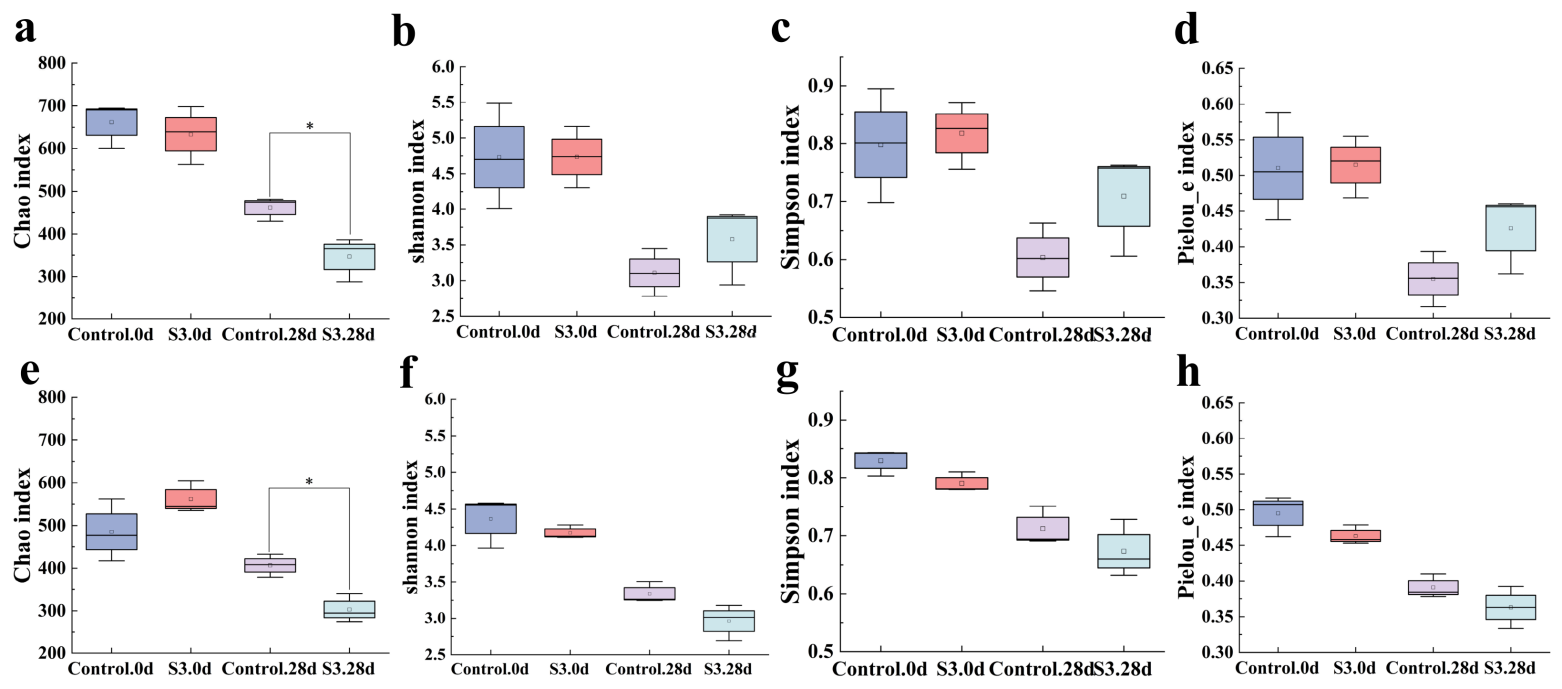
Effects of sethoxydim on rhizosphere fungal α-diversity in different foxtail millet cultivars. (**a**–**d**) Zhonggu 19; (**e**–**h**) Zhangzagu 10. Control, water-treated plots; S3, 337.5 g a.i./hm^2^ sethoxydim treatment; 0 d, before application; 28 d, 28 days after application. Asterisks (*) indicate significant differences between treatment and control by independent-samples *t*-test (*p* < 0.05).

Independent-samples t-tests showed significant differences in fungal α-diversity indices between the S3 treatment and the control at 28 days after application in both cultivars (*p* < 0.05). No significant differences were detected before application (0 d) in either cultivar (Figure 5). Chao1 index decreased significantly in Zhonggu 19 at 28 days after application (*p* < 0.05). Simpson and Pielou’s evenness indices increased. The Shannon index showed no significant change. Chao1, Shannon, Simpson, and Pielou’s evenness indices all decreased in Zhangzagu 10. This indicates a comprehensive reduction in fungal community richness, diversity, and evenness under stress.

#### 2.4.2. PCoA Analysis of Rhizosphere Fungal Communities

**Figure 6:**
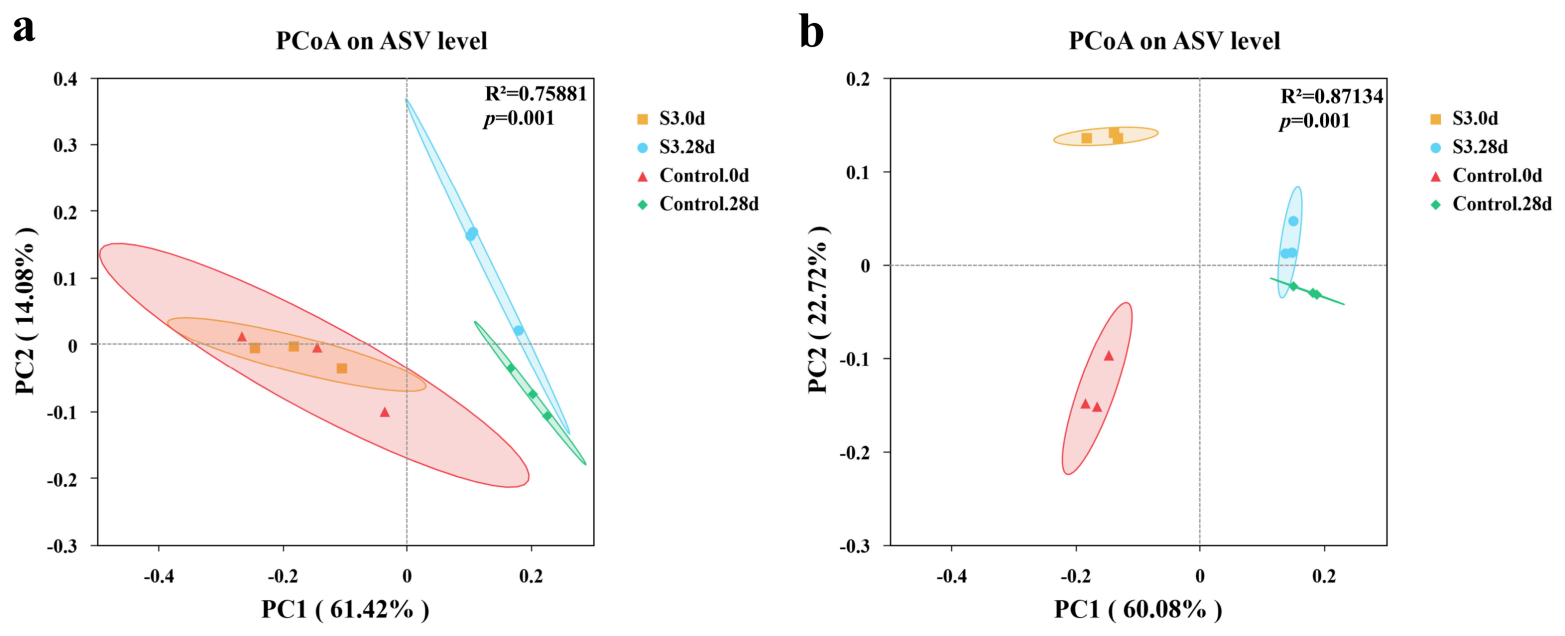
PCoA analysis of rhizosphere fungal communities after sethoxydim treatment (ASV level). (**a**) Zhonggu 19; (**b**) Zhangzagu 10. Control, water-treated plots; S3, 337.5 g a.i./hm^2^ sethoxydim treatment; 0 d, before application; 28 d, 28 days after application. PERMANOVA (Adonis) statistics for the overall four-group comparison are shown in the respective panels. Pairwise comparisons between Control and S3 at 28 d were not statistically significant (*p* > 0.05).

PCoA analysis based on ASV level showed structural changes in fungal communities before and after sethoxydim application in both cultivars (Figure 6). PERMANOVA (Adonis) revealed significant differences among all four groups in Zhonggu 19 (R^2^ = 0.759, *p* = 0.001) and Zhangzagu 10 (R^2^ = 0.871, *p* = 0.001). However, pairwise comparisons between Control and S3 at 28 d did not reach statistical significance in either cultivar (*p* > 0.05). PC1 and PC2 explained 61.40% and 14.07% of community variation in Zhonggu 19. The cumulative explained variation was 75.47%. The samples were obviously separated between 0 d and 28 d, and the 28 d treatment group was further separated from the control group without obvious overlap. PC1 and PC2 explained 60.08% and 22.72% of community variation in Zhangzagu 10. The cumulative explained variation was 82.80%. Although the 28d treatment group and the control group deviated from the 0 d region, the community composition shared certain similarities. A visual tendency for separation between 28 d Control and S3 could be observed in Zhonggu 19; however, this visual trend was less pronounced in Zhangzagu 10. Although the overall rhizosphere fungal community structure did not differ significantly between Control and S3 at 28 d in either cultivar, LEfSe differential abundance analysis identified multiple fungal biomarker taxa that responded to the S3 sethoxydim stress.

### 2.5. Relative Abundance of Rhizosphere Bacterial Communities at Phylum and Genus Levels

**Figure 7:**
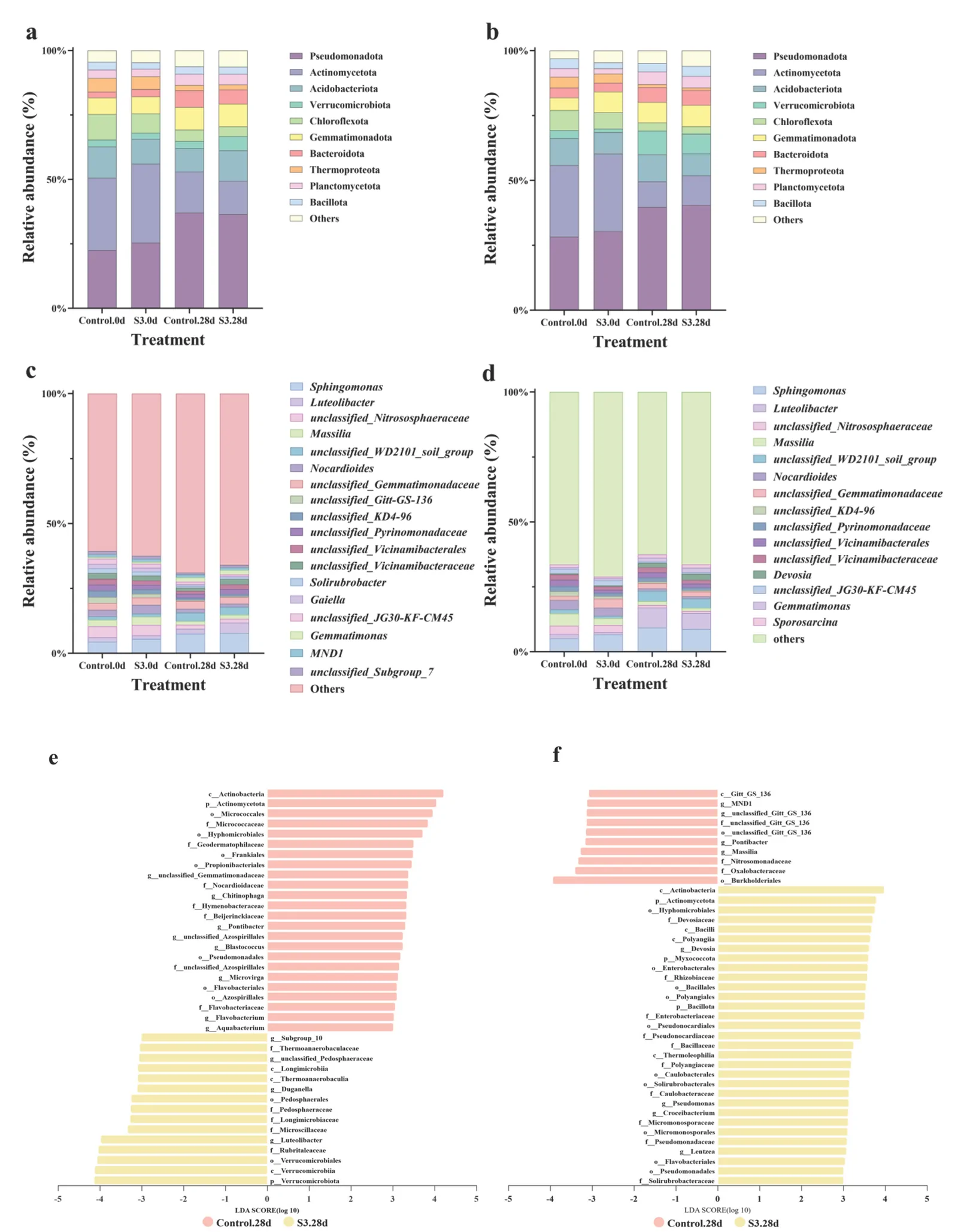
Relative abundance of rhizosphere bacterial communities at phylum and genus levels in two foxtail millet cultivars under sethoxydim treatment. (**a**) Phylum-level relative abundance for Zhonggu 19 (top 10 phyla); (**b**) phylum-level relative abundance for Zhangzagu 10 (top 10 phyla); (**c**) genus-level relative abundance for Zhonggu 19 (average relative abundance > 0.01); (**d**) genus-level relative abundance for Zhangzagu 10 (average relative abundance > 0.01). (**e**) LEfSe discriminative taxa (LDA > 3.0, *p* < 0.05) in Zhonggu 19; (**f**) LEfSe discriminative taxa (LDA > 3.0, *p* < 0.05) in Zhangzagu 10. Control, water-treated plots; S3, 337.5 g a.i./hm^2^ sethoxydim treatment; 0 d, before application; 28 d, 28 days after application.

At the phylum level, the dominant bacterial phyla, including Pseudomonadota, Actinomycetota, Bacteroidota, and Gemmatimonadota, were consistently present across treatments in both cultivars (Figure 7a,b). Observational trends in relative abundance were noted, such as an apparent decrease in Actinomycetota in Zhonggu 19 and an increase in Zhangzagu 10 under S3 treatment. However, these abundance shifts represent only observational patterns without formal statistical validation. LEfSe analysis (LDA > 3.0, *p* < 0.05) was therefore applied to identify discriminative biomarker phyla. In Zhonggu 19, Actinomycetota was enriched in the control group, whereas Verrucomicrobiota was enriched under S3 treatment (Figure 7e). In Zhangzagu 10, S3-treated samples showed enrichment of Actinomycetota, Myxococcota, and Bacillota (Figure 7f).

At the genus level, dominant bacterial genera with average relative abundance above 0.01 were analyzed, and cultivar-specific variations were evident (Figure 7c,d). Notable observational trends included apparent increases in *Luteolibacter* in Zhonggu 19 and *Devosia* in Zhangzagu 10 under S3 treatment. LEfSe analysis (LDA > 3.0, *p* < 0.05) was used to identify discriminative biomarker genera. In Zhonggu 19, *Luteolibacter*, *Duganella*, and unclassified_Pedosphaeraceae were enriched in the S3 group, while the control group was enriched in unclassified_Gemmatimonadaceae, *Chitinophaga*, *Pontibacter*, *Blastococcus*, and *Aquabacterium* (Figure 7e). In Zhangzagu 10, *Devosia*, *Pseudomonas*, *Croceibacterium*, and *Lentzea* were enriched under S3 treatment. By contrast, *Massilia*, *Pontibacter*, and MND1 were enriched in the control group (Figure 7f).

### 2.6. Relative Abundance of Rhizosphere Fungal Communities at Phylum and Genus Levels

**Figure 8:**
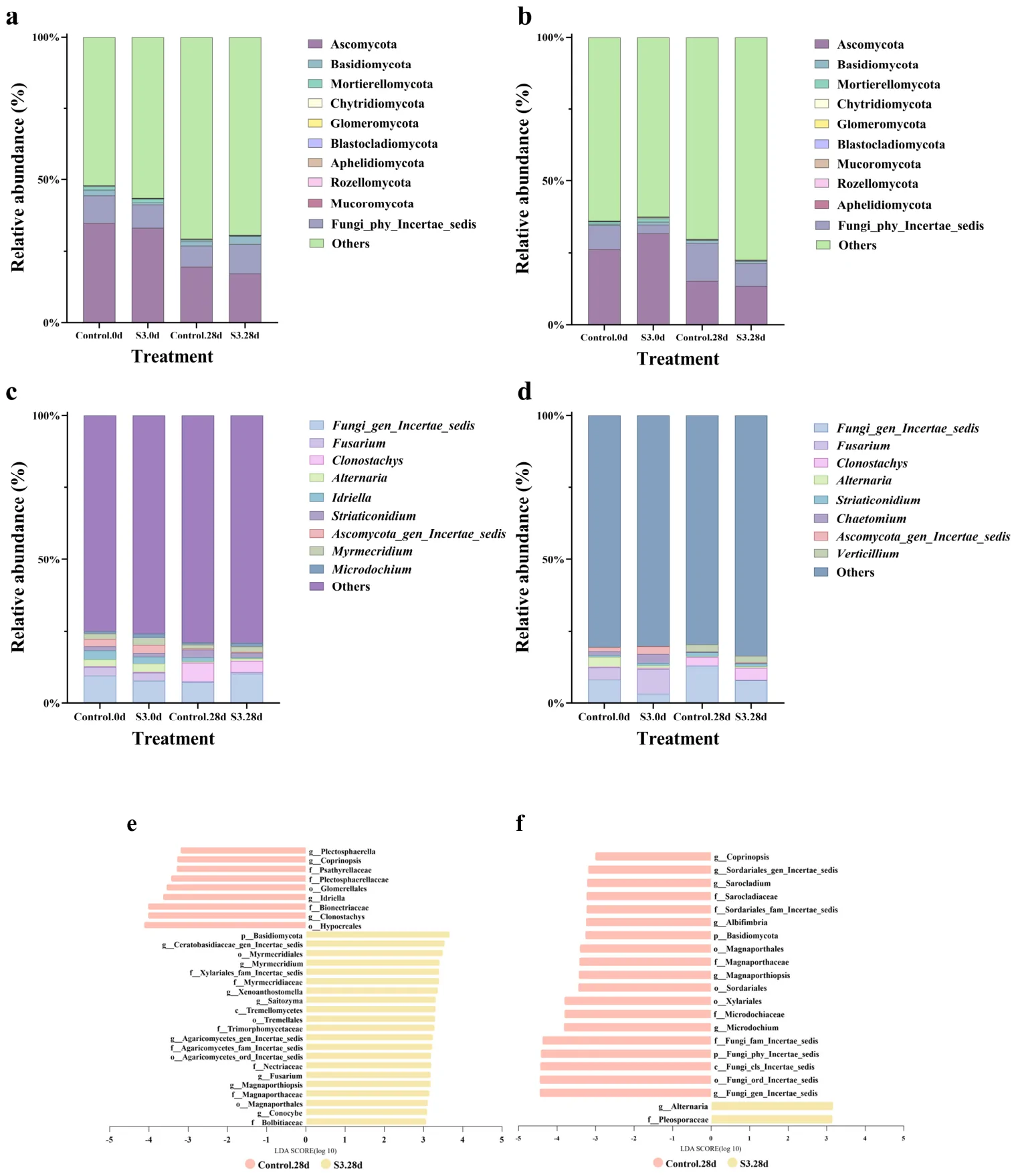
Relative abundance of rhizosphere fungal communities at phylum and genus levels in two foxtail millet cultivars under sethoxydim treatment. (**a**) Phylum-level relative abundance for Zhonggu 19 (top 10 phyla); (**b**) phylum-level relative abundance for Zhangzagu 10 (top 10 phyla); (**c**) genus-level relative abundance for Zhonggu 19 (average relative abundance > 0.01); (**d**) genus-level relative abundance for Zhangzagu 10 (average relative abundance > 0.01). (**e**) LEfSe discriminative taxa (LDA > 3.0, *p* < 0.05) in Zhonggu 19; (**f**) LEfSe discriminative taxa (LDA > 3.0, *p* < 0.05) in Zhangzagu 10. Control, water-treated plots; S3, 337.5 g a.i./hm^2^ sethoxydim treatment; 0 d, before application; 28 d, 28 days after application.

At the phylum level, the dominant fungal phyla, including Ascomycota, Basidiomycota, and Mortierellomycota, were consistently present across treatments in both cultivars (Figure 8a,b). Observational trends in relative abundance were noted, such as an apparent decrease in Ascomycota in both cultivars under S3 treatment. Basidiomycota showed an apparent increase in Zhonggu 19 but a decrease in Zhangzagu 10 under S3 treatment. Mortierellomycota decreased in both cultivars under treatment. However, these abundance shifts represent only observational patterns without formal statistical validation. LEfSe analysis (LDA > 3.0, *p* < 0.05) was therefore applied to identify discriminative biomarker phyla. In Zhonggu 19, Basidiomycota was enriched under S3 treatment (Figure 8e). In Zhangzagu 10, Basidiomycota was enriched in the control group (Figure 8f).

At the genus level, dominant fungal genera with average relative abundance above 0.01 were analyzed, and cultivar-specific variations were evident (Figure 8c,d). Notable observational trends included an apparent increase in *Fusarium* in Zhonggu 19 and a decrease in Zhangzagu 10 under S3 treatment. *Clonostachys* decreased in Zhonggu 19 but increased in Zhangzagu 10. *Alternaria* increased in both cultivars under treatment. *Chaetomium* increased markedly in Zhangzagu 10 but was not detected as a dominant genus in Zhonggu 19. LEfSe analysis (LDA > 3.0, *p* < 0.05) was used to identify discriminative biomarker genera. In Zhonggu 19, *Plectosphaerella*, *Clonostachys*, *Idriella*, and *Coprinopsis* were enriched in the control group. By contrast, *Fusarium*, *Magnaporthiopsis*, *Myrmecridium*, *Xenoanthostomella*, *Conocybe*, and *Saitozyma* were enriched under S3 treatment (Figure 8e). In Zhangzagu 10, *Sarocladium*, *Albifimbria*, *Magnaporthiopsis*, *Microdochium*, and *Coprinopsis* were enriched in the control group. Conversely, *Alternaria* was enriched under S3 treatment (Figure 8f). It should be noted that amplicon sequencing can only assign taxonomic affiliation at the genus level and cannot distinguish pathogenic from non-pathogenic strains within genera such as *Fusarium* and *Alternaria*; further isolation and pathogenicity assays are required to evaluate actual disease potential.

## 3. Discussion

Roots serve as the primary organ for water and nutrient uptake and represent the frontline for sensing herbicide stress in soil [25]. Herbicide-induced root growth inhibition has been widely documented; for instance, fomesafen significantly reduced root length and surface area in soybean seedlings [26]. In the present study, sethoxydim significantly reduced total root length, surface area, average diameter, and volume in foxtail millet seedlings, with effects intensifying at higher concentrations. The two cultivars showed distinct response patterns. Root growth in Zhonggu 19 was continuously suppressed, whereas Zhangzagu 10 exhibited significant inhibition only under S3, with some parameters increasing slightly under S2 [27,28]. Differences in root growth were observed between the two cultivars, which may reflect distinct tolerance patterns [29]. These root-level differences may subsequently influence rhizosphere biochemical processes, including the secretion and activity of soil enzymes.

Soil enzyme activity serves as a sensitive indicator of biological activity and nutrient cycling intensity [30]. Previous studies have demonstrated that various herbicides alter rhizosphere enzyme activities; for example, fomesafen inhibited urease and invertase [31], and tripyrasulfone initially inhibited urease before activating it [32]. In the present study, urease showed a decrease–increase–recovery pattern, consistent with transient disturbance and subsequent microbial adaptation [33]. The initial decrease may reflect direct toxicity to soil microorganisms [34], while microbial degradation subsequently reduces herbicide persistence [35]. The transient increase may represent a compensatory response to stress [36]. Sucrase was generally inhibited, suggesting prolonged disturbance to soil carbon metabolism [37], and catalase increased only during the early stage, indicating rapid redox recovery [38]. Recovery of urease and catalase appeared faster in Zhangzagu 10, possibly associated with better maintenance of root exudates [39]. Given that soil enzymes are primarily of microbial origin, these enzymatic patterns prompted us to examine whether the underlying microbial communities differed between cultivars.

The rhizosphere functions as a critical interface where plant roots, soil enzymes, and microorganisms interact dynamically. In this study, sethoxydim-induced root growth inhibition was accompanied by alterations in soil enzyme activities and shifts in the rhizosphere microbiome, suggesting an integrated response system. Below, we discuss these three components in an interconnected manner to elucidate the mechanisms underlying cultivar-specific tolerance to sethoxydim.

It should be noted that the following functional attributions of microbial taxa are inferred from taxonomic classification of amplicon sequencing and may not represent their actual in situ functional roles in the rhizosphere. Rhizosphere microbial communities play critical roles in plant nutrient acquisition, stress adaptation, and soil ecological balance [40]. These communities are sensitive indicators of herbicide-induced disturbances in the rhizosphere [41]. Previous studies have demonstrated that herbicides such as atrazine and mesosulfuron-methyl significantly alter rhizosphere microbial diversity and composition in various crop systems [42,43]. In the present study, sethoxydim altered α-diversity and community structure of both bacterial and fungal communities in foxtail millet rhizosphere, with cultivar-specific response patterns. Bacterial Chao1 index increased in Zhonggu 19. Shannon and Pielou’s evenness indices decreased. Fungal Chao1 index decreased significantly. Simpson and Pielou’s evenness indices increased significantly. This pattern may reflect the elimination of rare taxa or replacement by new rare taxa [44]. The total species number may increase. Community distribution becomes uneven. Bacterial Chao1 and Shannon indices decreased in Zhangzagu 10. Pielou’s evenness index increased significantly. Fungal Chao1, Shannon, Simpson, and Pielou’s evenness indices all decreased significantly. This may indicate that remaining community distribution tends toward uniformity after elimination of sensitive groups [45]. PCoA analysis confirmed significant shifts in bacterial and fungal community structures in both cultivars. The separation between treatment and control groups was greater in Zhonggu 19 than in Zhangzagu 10. This suggests a potentially stronger buffering capacity against sethoxydim stress in the latter cultivar under the tested conditions [46]. These inferences should be further verified in combination with community composition analysis.

Microbial responses to stress differed significantly at the phylum and genus levels. LEfSe analysis (LDA > 3.0, *p* < 0.05) identified distinct biomarkers in each cultivar. In Zhangzagu 10, the bacterial phyla Actinomycetota, Myxococcota, and Bacillota were enriched under S3 treatment. At the genus level, *Devosia*, *Pseudomonas*, *Croceibacterium*, and *Lentzea* were significantly enriched under S3 treatment. *Devosia* has been specifically documented as a plant growth-promoting bacterium [47]. Such bacterial taxa are known to facilitate nutrient acquisition and stress adaptation [48], and their interactions with root systems are fundamental to plant health under environmental stress [49]. In contrast, Zhonggu 19 showed enrichment of Verrucomicrobiota and the genera *Luteolibacter* and *Duganella* under S3 treatment, whereas the control group was enriched in Actinomycetota and genera such as *Chitinophaga* and *Blastococcus*.

At the fungal level, LEfSe analysis revealed that *Clonostachys* was significantly enriched in the control group of Zhonggu 19 (LDA = 4.02, *p* < 0.05). *Clonostachys* has been reported as a biocontrol agent with antagonistic effects against phytopathogenic fungi [50]. Although *Chaetomium* was not identified as a significant biomarker in the LEfSe analysis, it was observed as a dominant genus in Zhangzagu 10 and has been documented as a beneficial fungus involved in suppressing soil-borne pathogens [51]. In the sensitive cultivar Zhonggu 19, the loss of *Clonostachys* under S3 treatment coincided with the enrichment of *Fusarium* (LDA = 3.19, *p* < 0.05). However, it should be noted that the enrichment of *Fusarium* was based on relative abundance data from amplicon sequencing, which cannot confirm pathogenicity or absolute population changes. Therefore, claims regarding direct increases in disease risk are not supported by the present data. In Zhangzagu 10, fungal taxa enriched in the control group included *Microdochium*, *Sarocladium*, *Albifimbria*, and *Magnaporthiopsis*, while *Alternaria* was enriched under S3 treatment. The tolerant cultivar retained a more diverse assemblage of fungal taxa in the control state, suggesting greater community stability. Soil microbial communities are known to play important roles in suppressing soil-borne pathogens such as *Fusarium* [52,53].

While PERMANOVA indicated no significant overall community difference between Control and S3 at 28 d, LEfSe identified specific taxa responding to herbicide stress. This suggests selective pressure on particular taxa rather than wholesale community restructuring. In summary, our findings address the three hypotheses proposed in the Introduction. First, sethoxydim significantly inhibited root morphological development in Zhonggu 19, whereas Zhangzagu 10 maintained relatively stable root architecture, consistent with the hypothesis of cultivar-dependent sensitivity, though direct statistical comparison was not performed. Second, soil enzyme activities showed distinct patterns in the two cultivars, with Zhangzagu 10 exhibiting more resilient enzymatic profiles that may support nutrient cycling under stress. Third, LEfSe analysis revealed distinct bacterial and fungal biomarkers in each cultivar, with the tolerant cultivar recruiting taxa such as *Devosia*, *Pseudomonas*, and *Clonostachys* that are potentially involved in stress mitigation. These results collectively suggest that the apparently greater tolerance of Zhangzagu 10 under the tested conditions to sethoxydim is underpinned by coordinated adjustments in root physiology, enzymatic function, and microbial community structure.

These results provide a theoretical basis for screening herbicide-resistant foxtail millet cultivars and safe application of sethoxydim from a rhizosphere ecology perspective. Microbial sequencing was conducted only at 28 days after application. Dynamic data at intermediate time points are lacking. Rhizosphere sethoxydim residue and root exudate composition were not determined. Future research should integrate metabolomics and metagenomics. Longer-term field experiments are needed. Furthermore, separate one-way ANOVAs precluded formal testing of cultivar × dose interactions, which should be addressed in future studies using factorial or repeated-measures designs. These approaches will further elucidate the underlying mechanisms.

## 4. Materials and Methods

### 4.1. Experimental Materials

Two foxtail millet cultivars were used in this study: Zhonggu 19, a conventional cultivar provided by the Institute of Crop Sciences, Chinese Academy of Agricultural Sciences, and Zhangzagu 10, a hybrid cultivar developed by the Zhangjiakou Academy of Agricultural Sciences, Hebei Province. Both cultivars possess a basal level of tolerance to sethoxydim under field conditions; however, preliminary screening indicated that Zhangzagu 10 exhibits stronger tolerance, whereas Zhonggu 19 shows relatively weaker tolerance.

The herbicide used was sethoxydim (12.5% emulsifiable concentrate). It was manufactured by Shandong Xianda Agrochemical Co., Ltd., Binzhou China. The recommended field dose of sethoxydim (12.5% EC) for foxtail millet is 120 mL/mu, applied at a spray volume of 30 L/mu. Three application rates were tested: S1 (60 mL/mu, 0.5×), S2 (120 mL/mu, 1×, the recommended dose), and S3 (180 mL/mu, 1.5×). These correspond to 112.5, 225.0, and 337.5 g a.i./hm^2^, respectively. These three rates were selected to evaluate the dose-dependent effects of sethoxydim on foxtail millet and to identify a safe application range.

### 4.2. Experimental Design

The field experiment was conducted at the Dingxiang Experimental Station of Shanxi Agricultural University (38°35′08″ N, 112°53′15″ E), located at 022 Township Road, Dingxiang County, Shanxi Province, China. The study was carried out from May to September 2025. Meteorological data are presented in Table 1. The soil at the experimental site was a sandy loam with pH 8.44, available phosphorus 14.4 mg/kg, available potassium 151 mg/kg, organic matter 10.8 g/kg, total nitrogen 0.764 g/kg, total potassium 17.2 g/kg, total phosphorus 0.757 g/kg, and hydrolyzable nitrogen 75.8 mg/kg.

Seeds were sown on 2 June 2025. Both cultivars were subjected to identical treatments. The experiment followed a randomized complete block design with four biological replicates. Each plot measured 30 m^2^ (6 m × 5 m). Foliar spray application was performed at the three- to five-leaf stage on 6 July 2025 using a backpack sprayer with a flat-fan nozzle, delivering approximately 450 L/ha of spray solution under calm weather conditions between 9:00 and 11:00 a.m. (the dose settings are shown in Table 2). The control plots were sprayed with an equal volume of water (450 L/ha) under the same conditions. Sampling for root morphology and enzyme activity measurements was conducted at 0, 7, 14, 21, and 28 days after application. Microbiome sequencing was performed only at 0 and 28 days, representing the baseline and long-term response, respectively.

### 4.3. Analysis of Test Indicators and Methods

#### 4.3.1. Root Morphological Analysis

Four representative plants showing uniform growth, free of pests and diseases, and with similar plant height and leaf age were selected from each plot. Root systems were excavated from a soil volume of 20 cm × 20 cm × 20 cm. Roots were scanned using an Epson V700 scanner to obtain digital images. Root images were analyzed using WinRHIZO Pro 5.0 software. Total root length, total root surface area, average root diameter, and total root volume were calculated.

#### 4.3.2. Soil Enzyme Activity

Rhizosphere soil was collected by gently shaking the root system and brushing off the tightly adhering soil at each sampling time point (0, 7, 14, 21, and 28 days after application). The collected soil was air-dried at room temperature and passed through a 2 mm sieve. Soil enzyme activities were expressed per gram of air-dried soil. Urease activity was determined by the phenol-sodium hypochlorite colorimetric method. Absorbance was measured at 578 nm. Sucrase activity was determined by the 3,5-dinitrosalicylic acid (DNS) colorimetric method. Absorbance was measured at 508 nm. Catalase activity was determined by the potassium permanganate titration method. All methods followed Ge et al. (2018) [54].

#### 4.3.3. Rhizosphere Microbial Community Sequencing

Preliminary experiments showed that the high concentration (S3) caused the most significant stress effects. Cultivar differences were also most pronounced at this concentration. Long-term responses of foxtail millet rhizosphere microbiome to sethoxydim remain poorly understood. Response time patterns under pesticide stress were based on previous studies [55,56]. Two time points of 0 d (before spraying) and 28 d, as well as two treatments of Control and S3, were selected for sequencing analysis of the rhizosphere microbial community. Rhizosphere soil samples were collected at 0 days and 28 days after application to capture the immediate baseline and the long-term response of the microbial community to sethoxydim stress. Seedlings were carefully uprooted, large clods were removed, and soil adhering to the root surface (approximately 1–3 mm) was collected. This 1–3 mm zone is operationally defined as the rhizosphere, where root exudates and microbial interactions are most intense. This soil was sieved through a 2 mm mesh. Samples were flash-frozen in liquid nitrogen. They were stored at −80 °C. Samples were sent to Novogene Bioinformatics Technology Co., Ltd. (Beijing, China) for high-throughput sequencing. The company performed total DNA extraction, PCR amplification, library construction, and paired-end sequencing on the Illumina NovaSeq 6000 platform. Bacterial 16S rRNA gene V4 region was amplified using primers 515F (5′-GTGCCAGCMGCCGCGGTAA-3′) and 806R (5′-GGACTACHVGGGTWTCTAAT-3′). The Fungal ITS region was amplified using primers ITS5-1737F (5′-GGAAGTAAAAGTCGTAACAAGG-3′) and ITS2-2043R (5′-GCTGCGTTCTTCATCGATGC-3′). Raw data were quality-controlled and denoised using DADA2 by the company. Feature tables (ASV, 100% similarity) were constructed using the QIIME2 pipeline. Bacterial sequences were annotated against the SILVA database. Fungal sequences were annotated against the UNITE database. Sequencing was performed on both cultivars (Zhonggu 19 and Zhangzagu 10), with three biological replicates per treatment group (Control and S3) at both time points (0 and 28 days), totaling 24 samples. QIIME2 was used to calculate α-diversity indices (Chao1, Shannon, Simpson, and Pielou’s evenness). Principal coordinate analysis (PCoA) was performed based on Bray–Curtis distance. Fungal community data at the genus level were normalized across all samples. Fungal genera with relative abundance below 1% were classified as Others. Unclassified taxa with relative abundance below 1% were also classified as Others. PERMANOVA (Adonis, 999 permutations) based on Bray–Curtis distance was implemented in QIIME2 to test significant differences in community composition. LEfSe analysis was applied to identify discriminative biomarker taxa, with the threshold set as LDA > 3.0 and *p* < 0.05.

#### 4.3.4. Data Analysis

Data were processed using Microsoft Excel 2022 (Microsoft, Redmond, WA, USA). IBM SPSS Statistics 25 (SPSS Inc., Chicago, IL, USA) was used for statistical analysis. As the two cultivars are genetically distinct and were not directly compared across genotypes, one-way ANOVA was performed separately for each cultivar to evaluate the effects of herbicide dose on root morphological parameters and soil enzyme activities. For root morphology, the four plants sampled per plot were averaged to obtain a single plot-level value, with the plot serving as the unit of statistical replication (*n* = 4). Block effects were not included as a separate factor in the one-way ANOVA, as the analysis was designed to evaluate dose-dependent responses within each cultivar. Direct statistical comparison between cultivars was not performed. Therefore, claims regarding differential tolerance are interpreted as observational trends rather than statistically validated interactions. Duncan’s new multiple range test was used for multiple comparisons following one-way ANOVA. Significance was set at *p* < 0.05. Independent-samples t-tests were performed for α-diversity comparisons between groups. Significance was set at *p* < 0.05. Figures were generated using Origin 2024 (OriginLab, Northampton, MA, USA). GraphPad Prism 10 (GraphPad Software, San Diego, CA, USA) was also used. Data are presented as mean ± standard error (SE). Different lowercase letters denote significant differences among multiple-treatment comparisons at the same time point (*p* < 0.05). Asterisks (*) indicate significant differences between treatment and control based on independent-samples *t*-test (*p* < 0.05).

## 5. Conclusions

This study revealed the stress effects of sethoxydim on foxtail millet seedlings and cultivar differences across root morphology, soil enzyme activity, and rhizosphere microbial communities. Sethoxydim significantly inhibited root growth, with effects intensifying at higher concentrations. Zhangzagu 10 appeared to exhibit stronger tolerance and root plasticity relative to Zhonggu 19 under the tested conditions, showing marked inhibition only at 337.5 g a.i./hm^2^ (S3). Recovery of urease and catalase activities was also faster in Zhangzagu 10. Rhizosphere microbial community analysis further revealed that Zhangzagu 10 maintained greater bacterial community stability and LEfSe-identified enrichment of beneficial fungal taxa such as *Clonostachys*, whereas Zhonggu 19 showed significant shifts in fungal community composition under sethoxydim stress, including the LEfSe-identified enrichment of *Fusarium*. It should be noted that the enrichment of *Fusarium* was based on relative abundance data from amplicon sequencing, which cannot confirm pathogenicity. These findings suggest that sethoxydim should be applied at or below the recommended dose of 225.0 g a.i./hm^2^ (S2), particularly for susceptible cultivars such as Zhonggu 19, to minimize phytotoxicity and rhizosphere ecological disturbance. For fields planted with tolerant cultivars such as Zhangzagu 10, the recommended dose appears to carry a lower risk of adverse effects on root growth and beneficial soil microecology under the tested conditions. However, these dose recommendations are derived from preliminary experiments and require validation through additional field trials.

Several limitations should be acknowledged. First, microbial sequencing was conducted only at 0 and 28 days after application, lacking dynamic data at intermediate time points. Second, rhizosphere sethoxydim residue and root exudate composition were not determined. Third, this study was conducted at a single site during one growing season; therefore, the microbial community findings should be interpreted as preliminary mechanistic insights requiring validation through multi-site, multi-year experiments. Fourth, the study was conducted under field conditions with inherent environmental variability. Future research should integrate metabolomics and metagenomics approaches and include longer-term field experiments to further elucidate the underlying mechanisms.

## Figures and Tables

**Table 1:** Monthly mean temperature and total precipitation during the experimental period (May–September 2025) at the Dingxiang Experimental Station.

Month	Temperature Range (°C)	Total Precipitation (mm)
May	11–27	32.1
June	15–31	203.8
July	20–31	837.6
August	19–30	1049.6
September	12–25	152.2

Note: Temperature values are presented as daily minimum–maximum ranges. Precipitation data represent total monthly rainfall during the 2025 growing season.

**Table 2:** Setting scheme of different doses of sethoxydim/g a.i./hm^2^.

Treatment	Control	S1(1/2×)	S2(1×)	S3(3/2×)
Spraying amount	0	112.5	225.0	337.5

Note: The recommended dose of sethoxydim is 225 g a.i./hm^2^, and the spray volume is 450 L/hm^2^.

## Data Availability

The raw sequencing data presented in this study are openly available in the NCBI Sequence Read Archive (SRA) under BioProject accession number PRJNA1480967. The other data that support the findings of this study are available upon reasonable request from the corresponding author.
